# Oral health related quality of life of children and adolescents affected by rare orofacial diseases: a questionnaire-based cohort study

**DOI:** 10.1186/s13023-019-1109-2

**Published:** 2019-06-04

**Authors:** Lisa Friedlander, Ariane Berdal, Priscilla Boizeau, Brigitte Alliot Licht, Marie-Cécile Manière, Arnaud Picard, Olivier Azzis, Marie-Paule Vazquez, Corinne Alberti, Muriel De La Dure Molla

**Affiliations:** 1 Sorbonne Paris Cité, INSERM, Laboratoire ECEVE UMR1123, Université Paris Diderot, Paris, France; 2 Centre de Reference, Maladies Orales et Dentaires Rares, Hopital Rothschild, APHP, Paris, France; 3 Filière de Santé Maladies Rares TETECOU, Malformations Rares de la tête, du cou et des dents, Hôpital Necker, Paris, France; 4 Sorbonne Paris Cité, INSERM, Laboratoire de Physiopathologie Orale et Moléculaire, Université Paris Diderot, UMRS, 1138 Paris, France; 5 AP-HP, Unité d’Epidémiologie Clinique, Hôpital Robert Debré, Paris, France; 6 Sorbonne Paris-Cité, INSERM U1123 et CIC-EC 1426, Université Paris Diderot, Paris, France; 7 Centre de Competence, Maladies orales et Dentaires Rares, CHU de Nantes, Nantes, France; 8 Centre de Référence, Maladies orales et dentaires rares, Pôle de Médecine et Chirurgie Bucco-dentaires, Hôpitaux Universitaires de Strasbourg, Strasbourg, France; 9 Centre de Référence des Fentes et Malformations Faciales, Hôpital Necker, AP-HP, Paris, France; 10 Université Paris Descartes-Sorbonne Paris Cité, Paris, France; 11 Centre de Compétence des Fentes et Malformations Faciales, CHU de Rennes, Rennes, France; 12 INSERM UMRS1163 Bases Moléculaires et Physiopathologiques des Ostéochondrodysplasies, Institut Imagine, Necker, Paris, France

**Keywords:** Rare disease, Cleft, Oral manifestation, Teeth, Oral health-related quality of life (OHRQoL), Child, CHILD-OIDP

## Abstract

**Background:**

Rare diseases affecting the teeth, the oral cavity and the face are numerous, each of them present specific characteristics, and is a life-long condition. The aim of the study was to assess the association between Oral health-related quality of life (OHRQoL), and demographic characteristics, clinical and dental factors, and psycho-social characteristics to investigate that oral symptoms are not the main factors underlying a decrease in OHRQoL.

**Material and methods:**

We conducted a national cohort study in French centres for rare diseases (RD) specialized in orofacial diseases. The inclusion criteria were: to have received care in RD centres over the last 5 years (2012–2017) and to have been between 6 and 17 years of age on September 1, 2017. Patients were invited to answer a questionnaire composed of socio-demographic, clinical and dental questions, psychosocial questions and then fill in the Child-OIDP Index. At the end of the questionnaire, a free space was left for the patient to add a verbatim comment to provide qualitative data. Thematic analysis was used to analyze the verbatim answers.

**Results:**

Complete data were available for 110 patients. The sample included 44.5% boys and 55.5% girls. Ages ranged from 6 to 17 years old and 68.2% were between 6 to 12 years old and 31.8% were between 13 and 17 years old. Factor associated with a lower OHRQoL were: being a girl (*p* = 0.03), renouncement to dental care for financial reasons (*p* = 0.01), having syndromic disease (p = 0.01), having a problem with tooth shape and color (p = 0.03), feeling isolated, alone and different from other children (*p* = 0.003 and *p* = 0.02). Qualitative analysis highlighted very little recourse to psychological care and patients reported great anxiety and fear about the future.

**Conclusion:**

OHRQoL of children suffering from these diseases is impaired, especially from the psychosocial point of view but also from that of the course of treatment and access to care. There is a need to improve the legibility of care pathways and the financial coverage of treatments.

**Electronic supplementary material:**

The online version of this article (10.1186/s13023-019-1109-2) contains supplementary material, which is available to authorized users.

## Background

Rare dieases affecting the teeth, the oral cavity and the face are numerous although each specific disease is rare. Oral clefts and diseases such as multiple dental agenesis and amelogenesis imperfecta are examples of genetic conditions that affect head, neck and teeth in isolation or as part of a syndrome. These pathologies affecting the face have consequences on both appearance and oral function. Therapy involves a multidisciplinary team encompassing surgery, dental care, and speech therapy [1, 2]. Psychological management is needed because oral and general OHRQol are always impacted [2–4]. Indeed, with regard to facial clefts, surgical management begins in the first months of life and carries on throughout childhood, then from adolescence with orthodontic care and often in adult life with implants and prosthetic care [5].

More specifically, alteration of self-image was reported due to the facial malformation and handicap often generated [6, 7]. Educational integration, in terms of both social relations and school level, may be impaired by the disease [8].

To these difficulties are added financial issues. Dental prosthetic rehabilitation is poorly reimbursed in many countries such as France. In addition, travel costs are not supported and if the center of expertise is far from home, and costs can be important for families. Patients give up care for financial reasons [9].

Oral health-related quality of life has been extensively studied in the literature. It is now recognized that oral health cannot be dissociated from general health and therefore that oral health is a major component of the overall OHRQol but that oral parameters are not the only ones responsible for a lower general quality of life. According to the WHO, oral health affects people physically and psychologically, and not only influences how they grow, enjoy life, look, speak, chew, taste food and socialize, but also has an impact on their feelings of social well-being [10]. Our hypothesis was that elements like social and financial characteristics have a more negative effect on the oral health-related quality of life of young patients than the nature of the disease and its therapy. To validate this, we performed a quantitative and qualitative study to assess the association between OHRQol, and other factors such as demographic and psycho-social characteristics, clinical and dental factors, care course and renouncement of dental care. There are many scientifically validated tools available to study the oral health-related quality of life in children. In order to assess how these diseases affected patients’ ability to fully accomplish everyday tasks, the CHILD-OIDP questionnaire validated in French by Tubert-Jeannin S. and al. in 2005 [11] and initially validated by Gherunpong, S. and all [12] was chosen.

In addition, we performed a qualitative analysis based on a free-answer question asked at the end of the questionnaire, so that the patients could freely express feelings about their diseases and life.

Our objective was to provide the public authorities with reliable elements for implementing public health policies that are as close as possible to the real needs of children suffering from these diseases.

## Material and methods

### Study design

We conducted a national cohort study based on the RD network concerning the head, the neck and the teeth (TETECOU network).

Patients included in this network presented orofacial diseases. This network groups the 5 consultation centres included in this study.

### Recruitment

In this study were included patients followed in centers of expertise for oral dental and facial rare diseases. Patients can be separated in two groups: oral diseases (syndromic or not) without facial cleft (for example: amelogenesis imperfecta, dentinogenesis imperfecta, enamel-renal syndrome, ectodermal dysplasia, oligodontia ...) and rare orofacial diseases with presence of a facial cleft (eg Cleft lip and cleft palate, Pierre Robin syndrome, Goldenhar syndrome). Isolated and syndromic diseases were included in the study.

The director of the centres sent a list of patients treated in the centres over the last 5 years (2012–2017).

Inclusion criteria were: patients between 6 and 17 years old on September 1, 2017, who had been diagnosed with a cleft and/or a RD with or-dental manifestations at one of the expertise centres of the TETECOU network and consenting to participate in the study.

Exclusion criteria were patients who did not speak French, patients under legal protection and patients residing outside France (lack of comparability of health systems).

### Oral health-related quality of life

When applying the Child-OIDP, the children or their parents were asked to record a self-administrated version of the questionnaire. Patients recorded their oral problems (a list of dental problems was given in the questionnaire) during the last 3 months before they completed the questionnaire. The capacities of accomplishing certain functions were recorded (eating, speaking, cleaning mouth, sleeping, emotional status, smiling and social contact).

These 8 functions were rated on a 3-point scale, with possible answers ranging from “slight difficulty” to “medium difficulty”, to “great difficulty” corresponding to the values from 1 to 3 assigned to each answer.

For each task affected, the frequency was asked with possible answers ranging from “less than once a month” to “1 to 3 times a month”, to “3 or 4 times a month”, to “nearly every day”; response values ranging from 1 to 5 were assigned to each answer.

Each question was weighted in the same way; the maximum possible score was 100 points (worst possible result) and the lowest possible score was 0 point (best possible result). The mean values were calculated for each topic of the questionnaire as well as for the total score.

Higher scores thus indicated a lower OHRQol. The calculation of the score involved the multiplication of the severity and frequency for each function.

Patients were asked to complete a composite questionnaire composed of several parts: a socio-demographic part, a dental and clinical part, a psycho-social part then finally the CHILD-OIDP Index. Sociodemographic, clinical/dental, and psychosocial factors are described below.

### Potentially associated factors

#### Sociodemographic characteristics

Patient age and gender were considered. The method of social coverage was considered, depending on whether the patient had: no social security cover, social security alone, social security and additional coverage, “CMUc” (“CMUc” is a free complementary health cover designed to facilitate access to healthcare for people with low income living in France), “100% ALD” for their pathology (“100% ALD” is awarded if certain long-term conditions are considered “exonerating” for some dental care, except prosthetic care). As for school, the factors observed were type of schooling (traditional, or with personalized support), whether there was a learning delay, the level of education (kindergarten, primary, secondary or university). Regarding the financial aspect of care, the factors observed were: whether or not to give up dental care for the child, whether they had received help for care (from the city, local authorities or social security).

#### Clinical and dental characteristics

Regarding patient phenotype, we recorded the nature of the anomaly (concerning face, teeth or both), the diagnosis of syndrome association. All the children in the study suffered from dental problems due to their pathology. Some however had an oral cleft in addition. For this reason, we separated the patients in two groups: with or without the presence of a cleft. The following oral symptoms were checked: (in terms of absence or presence) toothache, tooth sensitivity, presence of caries, spaces between teeth due to no eruption of permanent teeth, falling decidual teeth, staining of teeth, altered tooth size and shape, badly positioned teeth, bleeding gums, swollen gums, mouth ulcers (aphtha type), bad breath, deformity of the mouth (cleft lip or palate), a problem of eruption of permanent teeth, a problem of absence of definitive teeth. Dental treatment was also recorded, especially prosthetic rehabilitation (such as removable or fixed prosthesis), and orthodontic appliances.

#### Psychosocial features

Psychosocial and psychic distresses were evaluated recording two items: feeling different from other children and feeling isolated at school.

Patient’s satisfaction about Communication between private doctors and doctors in hospital was assessed by asking the patients how they felt about this point.

### Data collection

Eligible persons were informed about the objectives and the modalities of the study. The questionnaire was filled electronically or on paper.

They were contacted by post. They received a letter containing the following elements:

- A letter describing the objectives and modalities of the study and inviting them to participate,
- A form to be returned if the participant wished to receive a questionnaire in paper form or online form.
- A pre-stamped envelope.

Patients had two ways to fill out the questionnaire: 1- by electronic means, with secure access to a dedicated site (secured by a personalized login and password). 2- by post: the respondents returned the form in the postage paid envelope and received the questionnaire in paper form and a pre-paid envelope.

In the event of non-response, a reminder letter (up letter) was sent one month after the first letter.

Data were recorded by one person (LF). The data management was performed to edit the data and verify the missing data.

### Statistical analysis

The primary outcome was the CHILD-OIDP score.

The statistical analyses were performed on participants for whom the CHILD-OIDP and the potentially associated factors were recorded exhaustively.

Data were expressed in medians (1st; 3rd quartile), minimum and maximum for non-Gaussian continuous variables and in numbers (percentages) for categorical variables.

Bivariate and multivariate analyses were carried out to identify factors associated with the CHILD-OIDP using quantile regression. Beta coefficient and 95% confidence intervals were calculated.

All the data were analyzed using SAS software (version 9.4; SAS Institute, Cary, NC, USA).

All the statistical tests were bilateral with a significance level of 5%, which means that a *p*-value< 5% was considered significant.

#### Bivariate analyses

As a preliminary step in the multivariate analyze, we performed bivariate analyses to examine the differences of the mean CHILD-OIDP score across subgroups of potentially associated factors.

#### Multivariate analyses

Significant factors at the 20% bivariate threshold were selected for the multivariate model. An automatic stepwise selection technique called “stepwise” was used. It allowed obtaining a model with only significant factors at 5%, adjusted for age in 2 classes, gender and whether the disease was syndromic or not.

We tested the adequacy of the regression by graphically verifying that the assumptions of homoscedasticity and normality of the estimation errors were satisfied by building a scatter plot of the estimation errors against the estimated CHILD-OIDP scores and a quantile-quantile plot. As the distributions were not normally distributed, the quantitative data were summarized by the median (1st, 3rd quartile) and the minimum and maximum were also given.

### Qualitative methodology

At the end of the questionnaire, a free space was left for the patient to add a verbatim if they wished. No specific question was asked; the order was simply to express freely what they wanted to say about the disease, the care course, their life or their children’s life.

An inductive approach using thematic analysis was used to manually analyze the verbatim answers [13–15]. This method allowed organizing thematic and to identify and organize patterns. The objective was to precisely analyze the whole dataset. Topics were chosen for their prevalence and their importance in relation with the research question.

## Results

Five expertise centres participated in the study. Two specialized in maxillo-facial surgery and three in oral surgery.

**Fig. 1:**
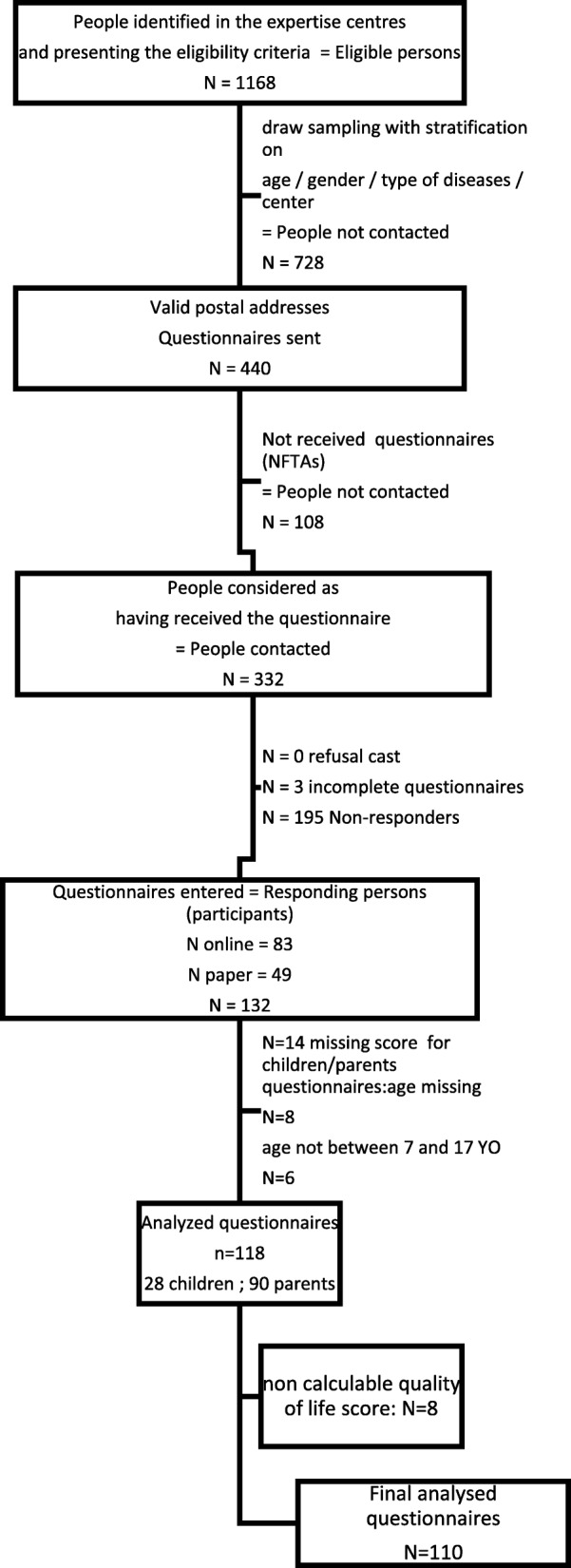
Study flow chart

A list of 1168 patients was obtained from the centres. A sample was obtained after eliminating patients not living at the declared address, and by stratifying patients as a function of age, gender and disease type (cleft versus dental rare RD). The final analysis was performed on 440 children between 6 and 17 years old (Fig. 1). Overall, 108 patients did not receive the questionnaire because their address had changed, leading to the inclusion of 132 patients in the survey. Complete data (people whose score was calculable and who respected inclusion criteria) were available for 110 patients.

The sample included 49 boys (44.5%) and 61 girls (55.5%). Ages ranged from 6 to 17 years old and 75 were between 6 to 12 years old (68.2%) and 35 were between 13 and 17 years old (31.8%).

Regarding the children’s social coverage, 103 (94.5%) of them were covered by social security and private health insurance. In this sample, 54 (55.1%) of the children benefited from 100% coverage by social security (“100% ALD”).

Regarding the distribution by type of disease, 51 (51.5%) of the children in this sample presented an oral cleft and 48 (48.5%) of them had an isolated dental disease. There were 32 (32.3%) of children affected by syndromic disease.

The OHRQol score, CHILD-OIDP, ranged from 6.7 to 80 with a median of 20.8 (Table 1).

**Table 1:** Demographic and social description of the sample

Variables	Description
Gender	*N* = 110
Male	49 (45%)
Female	61 (55%)
Age subgroups	N = 110
6–12 YO	75 (68.2%)
13–17 YO	35 (31.8%)
Social coverage	*N* = 109
National coverage without private insurance	2 (1.8%)
“CMUc”	4 (3.7%)
National coverage with private insurance	103 (94.5%)
« 100% ALD »	*N* = 98
No	44 (44.9%)
Yes	54 (55.1%)
type of disease	*N* = 99
Oral Cleft	51 (51.5%)
Dental diseases	48 (48.5%)
Syndromic disease	N = 99
Syndromic	32 (32.3%)
Non-syndromic	67 (67.7%)
Index Child OIDP	N = 110
Median (Q1; Q3)	20.8 (10.0;35.0)
Min; Max	6.7;80.0

### Bivariate analysis

Sociodemographic variables, clinical, medical and psychosocial variables were compared to the OHRQol scoring. Regarding sociodemographic variables, being a girl was significantly associated with a lower OHRQol (p = 0.03). Foregoing dental care for financial reasons was also associated with a lower OHRQol (p = 0.01) (Table 2).

**Table 2:** CHILD-OIDP score by socio-demographic characteristics (*N* = 110)

Socio-demographic characteristics	n	%	CHILD-OIDP score		Beta [95% CI]	*P* value
			Median (Q1; Q3)	Min; max		
Gender						
Male	49	44.5	15.0 (7.5; 29.2)	6.7; 66.7	0.0	-.
Female	61	55.5	24.2 (11.7; 40.0)	6.7; 80.0	9.2 [0.7; 17.7]	**0.03**
Age						
6–12 years old	75	68.2	20.8 (10.0; 35.0)	6.7; 80.0	0.0	–
13–17 years old	35	31.8	22.5 (9.2; 43.3)	6.7; 61.7	1.7 [−9.2; 12.6]	0.76
Education level						
Pre-school/Primary School	72	65.5	20.8 (10.8; 35.0)	6.7; 80.0	0.0	–
College/University	38	34.5	21.3 (8.3; 40.0)	6.7; 72.5	1.7 [− 8.3; 11.7]	0.74
Type of school						
Traditional	89	82.4	17.5 (9.2; 30.8)	6.7; 66.7	0.0	.
Personalized	19	17.6	29.2 (16.7; 61.7)	6.7; 80.0	11.7 [−9.6; 33.0]	0.28
Academic delay						
No	94	85.5	17.9 (9.2; 33.3)	6.7; 80.0	0.0	.
Yes	16	14.5	33.0 (21.3; 52.1)	9.2; 77.5	13.4 [−2.0; 28.8]	0.09
100% ALD						
No	44	44.9	19.6 (9.2; 33.8)	6.7; 66.7	0.0	.
Yes	54	55.1	25.0 (11.7; 40.0)	6.7; 80.0	5.0 [−4.5; 14.5]	0.30
Renouncement to dental care						
No	97	89.0	20.0 (9.2; 33.3)	6.7; 80.0	0.0	.
Yes	12	11.0	40.9 (17.9; 44.6)	6.7; 62.5	20.0 [4.1; 35.9]	**0.01**
Financial help received for dental care						
No	96	91.4	20.0 (9.2; 34.6)	6.7; 80.0	0.0	.
Yes	9	8.6	31.7 (25.0; 50.8)	10.0; 77.5	11.7 [−9.3; 32.7]	0.27

Bold indicates *p* values under 0.05.

For the clinical and medical variables, syndromic disease was significantly associated with a worse OHRQol (*p* = 0.01). Having a problem of tooth shape and color was the only dental factor, among all the oral variables studied, associated with poorer OHRQol (*p* = 0.03) (Table 3).

**Table 3:** CHILD-OIDP score by clinical and dental characteristics (N = 110)

Clinical and dental characteristics	n	%	CHILD-OIDP score		Beta [95% CI]	*P* value
			Median (Q1; Q3)	Min; max		
Syndromic disease						
Yes	32	32.3	33.0 (16.3; 44.6)	6.7; 77.5	15.0 [3.7; 26.3]	**0.01**
No	67	67.7	16.7 (9.2; 30.0)	6.7; 80.0	0.0	
Type of disease						
Disease with Oral cleft	51	51.5	26.7 (13.3; 39.2)	6.7; 80.0	0.0	.
Dental disease	48	48.5	16.3 (9.2; 32.5)	6.7; 77.5	−10.0 [− 19.8; −0.2]	0.05
Removable prosthesis						
No	101	91.8	20.8 (10.0; 35.0)	6.7; 80.0	0.0	.
Yes	9	8.2	30.0 (13.3; 41.7)	6.7; 44.2	9.2 [−14.3; 32.7]	0.44
Fixed prosthesis						
No	100	90.9	21.7 (10.4; 35.9)	6.7; 80.0	0.0	.
Yes	10	9.1	14.6 (7.5; 20.8)	6.7; 66.7	−5.9 [−17.4; 5.6]	0.31
Orthodontic treatment						
No	64	58.2	16.7 (8.8; 33.4)	6.7; 77.5	0.0	.
Yes	46	41.8	24.2 (10.8; 43.3)	6.7; 80.0	7.5 [−2.5; 17.5]	0.14
Painful teeth						
No	75	70.1	20.0 (9.2; 34.2)	6.7; 77.5	0.0	.
Yes	32	29.9	24.2 (12.5; 44.6)	6.7; 80.0	4.2 [−9.1; 17.5]	0.53
Sensitive teeth						
No	58	56.3	20.4 (9.2; 34.2)	6.7; 77.5	0.0	
Yes	45	43.7	20.8 (13.3; 39.2)	6.7; 80.0	0.0 [−9.4; 9.4]	>.99
Decays						
No	83	76.9	21.7 (10.0; 35.0)	6.7; 77.5	0.0	.
Yes	25	23.1	20.8 (9.2; 41.7)	6.7; 80.0	−0.9 [−13.0; 11.2]	0.88
Space between deciduous teeth did not fill						
No	40	38.1	21.7 (9.2; 37.5)	6.7; 77.5	0.0	.
Yes	65	61.9	19.2 (10.8; 31.7)	6.7; 80.0	−2.5 [−13.3; 8.3]	0.65
Deciduous teeth falling						
No	44	40.4	17.1 (7.9; 32.5)	6.7; 62.5	0.0	.
Yes	65	59.6	24.2 (11.7; 39.2)	6.7; 80.0	6.7 [−2.4; 15.8]	0.15
Colored teeth						
No	64	61.0	21.3 (10.0; 37.5)	6.7; 72.5	0.0	.
Yes	41	39.0	21.7 (10.0; 30.8)	6.7; 77.5	0.0 [−9.9; 9.9]	>.99
Anomaly of size and shape of teeth						
No	33	32.7	14.2 (7.5; 24.2)	6.7; 51.7	10.0 [1.0; 19.0]	
Yes	68	67.3	24.6 (12.5; 42.5)	6.7; 80.0	0.0	**0.03**
Teeth alignment problems						
No	21	19.3	15.8 (9.2; 35.0)	6.7; 66.7	0.0	.
Yes	88	80.7	21.7 (10.8; 36.7)	6.7; 80.0	5.9 [−8.1; 19.9]	0.41
Gum bleeding						
No	81	74.3	18.3 (9.2; 31.7)	6.7; 72.5	0.0	
Yes	28	25.7	26.7 (13.0; 43.4)	6.7; 80.0	5.9 [−6.6; 18.4]	0.35
Swollen gums						
No	89	85.6	17.5 (9.2; 34.2)	6.7; 77.5	0.0	
Yes	15	14.4	25.0 (13.3; 44.2)	6.7; 80.0	7.5 [−6.2; 21.2]	0.28
Mouth ulcers, aphta						
No	81	75.0	18.3 (9.2; 33.3)	6.7; 77.5	0.0	
Yes	27	25.0	24.2 (13.3; 45.0)	6.7; 80.0	5.9 [−8.7; 20.5]	0.42
Bad breath						
No	76	70.4	21.7 (9.2; 37.5)	6.7; 80.0	0.0	
Yes	32	29.6	20.4 (12.1; 34.6)	6.7; 77.5	−0.9 [−10.6; 8.8]	0.85
Mouth deformation (cleft)						
No	68	63.6	17.9 (8.8; 33.0)	6.7; 77.5	0.0	
Yes	39	36.4	24.2 (11.7; 43.3)	6.7; 80.0	5.9 [−4.5; 16.3]	0.26
Anomaly of eruption of definitive teeth						
No	43	40.6	20.0 (8.3; 44.2)	6.7; 66.7	0.0	
Yes	63	59.4	21.7 (10.8; 30.0)	6.7; 80.0	1.7 [−11.7; 15.1]	0.80
Lack of definitive teeth						
No	51	50.5	20.8 (10.0; 33.3)	6.7; 77.5	0.0	
Yes	50	49.5	19.2 (9.2; 40.0)	6.7; 80.0	−0.8 [−11.0; 9.4]	0.88

Bold indicates *p* values under 0.05.

Regarding psychosocial variables, feeling isolated and alone because of the disease was significantly associated with a lower OHRQol (*p* = 0.003). Feeling different from other children was also significantly associated with a lower OHRQol (*p* = 0.02).

On the other hand, the quality of relations between hospital and private medicine did not seem to have a significant influence on the OHRQol of patients. (Table 4).

**Table 4:** CHILD-OIDP score by psychosocial characteristics (N = 110)

Psychosocial characteristics	n	%	CHILD-OIDP score		Beta [95% CI]	*P* value
			Median (Q1; Q3)	Min; max		
Feeling of being sidelined in life						
No	72	69.9	16.7 (8.3; 28.8)	6.7; 77.5	0.0	
Yes	31	30.1	36.7 (16.7; 51.7)	6.7; 80.0	20.0 [6.8; 33.2]	**0.003**
Feeling different						
No	38	36.9	16.7 (8.3; 25.0)	6.7; 77.5	0.0	
Yes	65	63.1	27.5 (13.3; 44.2)	6.7; 80.0	10.8 [1.6; 20.0]	**0.02**
Relationship between hospital and private medicine						
Good	67	60.9	20.0 (9.2; 30.0)	6.7; 77.5	0.0	.
Bad	22	20.0	19.2 (11.7; 45.0)	6.7; 80.0	1.7 [−15.2; 18.6]	0.84
Doesn’t know	21	19.1	28.3 (9.2; 40.0)	6.7; 61.7	8.3 [−5.2; 21.8]	0.23

Bold indicates *p* values under 0.05.

**Multivariate analysis.**

A threshold of 20% in the bivariate model was chosen for the variables selected for the multivariate model (gender, academic delay, renouncement to dental care, syndromic disease, type of disease, orthodontic treatment, deciduous teeth, being sidelined in life, feeling different).

Multivariate analysis showed that the variables associated with a poorer OHRQol, adjusted on age in 2 classes: 6–12 years old / 13–17 years old, gender: male / female, syndromic disease or not, were: diagnosis of syndromic disease (*p* = 0.04), dental disease without the presence of a facial cleft (*p* = 0.002), problems with the shape and size of the teeth (*p* = 0.005) and feeling sidelined and alone (*p* = 0.003) (Table 5).

**Table 5:** CHILD-OIDP score – multivariable model (*N* = 110)

Clinical and dental characteristics	Beta [95% CI]	*P* value
Gender		
Male	0.0	-.
Female	4.2 [−2.8; 11.1]	0.23
Age		
6–12 years old	0.0	–
13–17 years old	−0.9 [− 8.9; 7.1]	0.82
Syndromic disease		
Yes	8.4 [0.4; 16.3]	**0.04**
No	0.0	.
Type of disease		
Disease with facial cleft	0.0	**.**
Dental disease	−10.8 [−17.6; −4.0]	**0.002**
Problem of size and shape of teeth		
Yes	9.2 [2.9; 15.4]	**0.005**
No	0.0	.
Feeling of being sidelined in life		
Yes	20.0 [6.9; 33.1]	**0.003**
No	0.0	.

Bold indicates *p* values under 0.05.

### Qualitative analysis

The verbatim responses were analyzed for qualitative appreciation of patient illness. 36% (40/110) of the patient answered. Thematic analysis was used to analyze the interview data [16]. All the verbatim responses can be found in the Additional file 1.

**Fig. 2:**
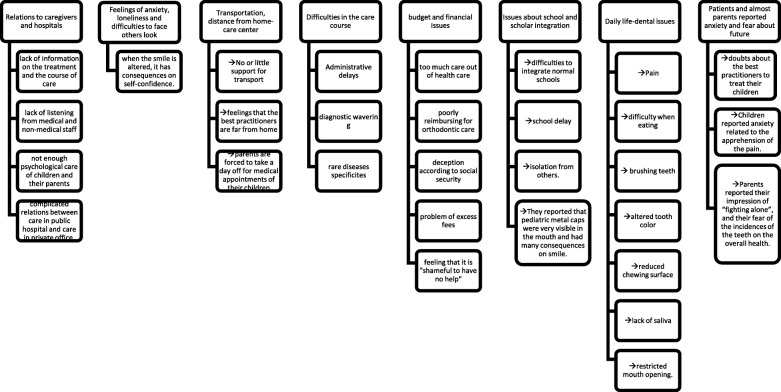
Categories and sub-categories of qualitative analysis topics

The topics addressed in these verbatim responses can be classified according to the following topics (Fig. 2): patients reported a lack of information on treatment and the course of care, and a lack of listening from medical and non-medical staff. Insufficient psychological care of children and their parents was reported. They also reported complicated relations between care in public hospital and care in private establishments (Topic 1).

Patients and their parents reported feelings of anxiety, loneliness and difficulties in facing the gaze of others. They expressed that spoiled smiles damage self-confidence (Topic 2).

Patients and families complained about transport, stating that the best practitioners were far from home. Parents were forced to take a day off for the medical appointments of their children (Topic 3).

Patients reported many difficulties in the care course (Topic 4).

School and academic integration appeared to be bad. They reported learning delays, difficulties in becoming integrated in normal schools, and isolation from others (Topics 5). They also reported that pediatric metal caps were very visible in the mouth and had many impacts on smiling. Patients reported several daily life-dental issues: pain, difficulty when eating, brushing teeth, altered tooth color, reduced chewing surface, lack of saliva, restricted mouth opening (Topics 6).

A large proportion of patients reported budget and financial issues. Patients reported their deception according to social coverage: problem of excess fees (treatments too expensive and not enough reimbursed), feeling that the lack of financial assistance was not acceptable (Topic 7).

The patients and most parents reported anxiety and fear about the future. They had doubts about the most competent practitioner to treat their children. Children reported anxiety related to the fear of pain. Parents reported their impression of “fighting alone”, and their fear of the impact of teeth on overall health (Topic 8).

## Discussion

Our hypothesis, verified here, was that medical and oral alteration was not the only factor responsible for harming children’s OHRQol.

For example, the bivariate analysis showed that being a girl was significantly associated with a lower OHRQol (*p* = 0.03). This result corroborated several studies on the OHRQol of patients with clefts or facial alteration [17]. On the other hand, several studies with smaller cohorts did not show this difference [18, 19].

The physical and social deterioration of these diseases affecting the orofacial sphere may be more difficult for young and teenage girls who may be under significant social and aesthetic pressure.

Having a syndromic disease was the second reason for poor OHRQol (*p* = 0.01).

The syndromic forms, more serious and disabling because they were sometimes associated with other motor and / or intellectual handicaps, required more intensive overall care [20].

The study has shown in the multivariate model that having an isolated dental disease (without the presence of a facial cleft) was associated with a poorer OHRQol (*p* = 0.002) than those who suffered additionally from a facial cleft. Dental rare diseases can concern anomalies of: number of teeth (agenesis, anodontia, oligodontia, supernumerary teeth), size and shape of the teeth (microdontia, conical teeth...), structure of the teeth (amelogenesis imperfecta, enamel hypoplasia, hypomineralization, dentinogenesis imperfecta, dentinal dysplasia gingival and parodontal diseases (gingival overgrows, gingival fibromatosis, periodontitis), eruption, of the teeth (retainend teethn delay of eruption of the teeth …). These diseases have functional repercussions but also aesthetic and social because the appearance can be largely altered. [6, 21–23]. However, this result appeared surprising since having a facial cleft seemed to be more disabling, especially if it is syndromic. Nevertheless, the diagnosis of facial clefts was prenatal. Our study led us to form the hypothesis that short and long-term treatments were well established and organized around a multidisciplinary team including psychological care. On the contrary, this study showed that difficulties related to funding and care pathways appear more present in patients with dental diseases (shape and color alteration of teeth, orthodontics treatment) (Table 3). The qualitative analysis also revealed that patients and their parents reported daily life-dental issues: pain, difficulty when eating, brushing teeth, altered tooth color, reduced chewing surface, lack of saliva, and restricted mouth opening. These issues did not appear in the quantitative analysis.

The third issue associated with a lower OHRQol was the renouncement of dental care (*p* = 0.01). This renouncement may be explained by many reasons such as financial cost, easy access to care, and difficulty to choose an expert practitioner to perform the complex treatments required. Oral care for children suffering from rare dental diseases (oligodontia, ectodermal dysplasia, for example) most often required multidisciplinary treatments include orthodontics and sometimes orthognathic surgery, conservative care, removable prosthesis at first and conventional fixed dental prosthesis and implant prosthetic rehabilitation at the end of growth [24–26]. The treatments are long and are not or only partly covered by health insurance. Moreover, treatments become more complex and expensive with age. Therefore, there is in increasing correspondence between the renouncement of care and worsening OHRQol (Table 2). Qualitative analysis revealed that cost was an issue that worried parents. In France, the socialized share of expenses incurred by the prosthetic and orthodontic rehabilitation of children and adults with rare orofacial diseases is not greater than for healthy patients, except for implants (without prosthesis) in the case of ectodermal dysplasia. No care scheme is currently planned for patients suffering from diseases affecting the oral sphere and whose lifelong care will be long and expensive. For all these reasons, we propose that a “rare orofacial disease care package” would be a good health policy for improving the OHRQol of patients with dental diseases.

The qualitative analysis also showed that the renouncement of dental care increases with the rarity of the disease. Parents and patients mentioned difficulties regarding diagnosis and surgery [27].

Feeling isolated, alone and different from other children was significantly associated with a lower OHRQol in the quantitative analysis. In the qualitative analysis, parents reported that their children were anxious about their future and their daily lives. Their appearance was sometimes greatly altered, and the gaze of others is a source of discomfort and anxiety. Lack of self-confidence, fear of smiling and the feeling of being beautiful appeared to be important for these young people. It is important to systematically make available help and psychological support during childhood and during the transition from pediatric to adult medical treatment. The literature showed that appearance and speech were the most frequently discussed points for children with facial clefts. Individuals with facial clefts may struggle with the psychological and social sequels of having differences in their appearance and speech [28].

This lack of psychological care has been accompanied, according to the qualitative analysis of the verbatim comments, by poor communication on the part of doctors. Children and their families reported anxiety about the future and the lack of responses to their questions about future treatments. This study has shown the interest of the identification of psychological factors that could be used to improve children’s self-esteem and OHRQol.

Very few studies have mixed quantitative and qualitative analyzes regarding rare orofacial diseases [29, 30]. The existing literature with lip and/or palate cleft has focused almost exclusively on the medical aspects of care rather than patient experience, on objective measures rather than subjective self-perceptions, and on deficits rather than strengths.

The literature showed that the OHRQol of children with oral disease was much worse than in the general population [12, 31, 32]. The median CHILD-OIDP score was 20.08/100 while for the general population it is between 2 and 8 [11, 33]. The Child-OIDP score had the advantage of combining varied performances ranging from daily life to social relations. It had been shown in the literature that neither self-administration nor accompaniment by an investigator affect the results of questionnaires [34]. Thus, the fact that children responded with their parents, when they were not able to do it alone, did not limit the correct interpretation of the answers [35, 36].

### Limits

In terms of bias, it is questionable whether those who responded to the study feel better or worse than those who did not respond. This can be seen in the qualitative analysis which may appear in some points as a list of grievances in which patients finally have a place to freely express their feelings and their distress about the management of the disease and the course of care. We did not analyze who did not answer, which could be a source of selection bias. However, the results corroborated those in the literature and, thanks to the qualitative analysis, provided much information on the feelings of patients, little studied so far.

Another point which may be discussed concerns the sample size and therefore the external validity of the findings and their representativeness. This study brings one of the largest samples on these topics studied in France to date. These data are the first on the management of these patients in a country where many political efforts have been made on rare diseases. This sample is interesting in relation to the initial population, strictly drawn and randomized, because it keeps the initial stratification (on gender, sex, type of disease) and the findings verify our initial hypothesis.

Our objective was not to conduct a study measuring statistical inference or to bring prognostic factors. The aim of this study was to better understand management of patients and how it could be improved. The results show inequalities in access to care with a lack of psychological support and financial coverage for their care. This data will be interesting to compare with other countries in the future.

With regard to the large number of diseases studied, they have in common the oral and social handicaps in their different syndromic composition’s. These disabilities produce significant social inequalities. The stratification on age, sex and type of disease in the sample are clearly shown in the results by showing sub-groups of patients less well managed on both the medical and psychological sides.

With the contribution of the analytical results from the qualitative study, this study will be an important basis for other intervention studies.

### Strengths

Although we did not carry out real mixed-method research, our study was greatly enriched by the analysis of the patients’ and parents’ verbatim comments.

The qualitative analysis highlighted topics that have not been explored through quantitative analysis, such as the fear of the future, shared by children and parents, the perception of feeling alone with the disease, from the standpoint of the care path and the overall integration of the child. This qualitative analysis will provide new topics and variables for future quantitative studies.

The lives of these children are clearly affected by their illnesses, but as the results of the quantitative analysis, and more precisely those of the qualitative analysis, have shown, the purely dental and oral parameters did not stand out as being clearly significantly related to the OHRQol as expressed in the study.

These conclusions are interesting because, so far, very few studies have shown the possible associations between psychosocial and socioeconomic factors related to the OHRQoL affected by these pathologies. Oral and dental explanations are often given without taking into consideration the whole existence of the child and their parents. What does it mean to be a carrier of a RD that affects appearance, whose necessary care is very expensive and poorly reimbursed whose care course, is chaotic?

In this study, all the results converged towards a need to improve the legibility of care pathways and financial coverage of treatments. Public policies must improve existing procedures so that no child with a rare disabling illness at the level of function and social acceptance is obliged to give up the care that is essential to their well-being and OHRQoL This study managed to provide several answers to these questions and suggest orientations in terms of health policy.

## Conclusion

Although many efforts have been made in recent years regarding policies designed to improve access to the diagnosis and care of patients with RD in France, care pathways remain very complex for patients and their families. Financial support for necessary and oral care is still a big problem for families who must give up certain of its essential aspects.

Public policies must improve procedures so that no child with a rare disabling illness at the level of function and social acceptance has to give up the care essential to their well-being and OHRQoL. This study managed to provide some answers to these questions and to suggest orientations in terms of health policy. In addition to working for a better financial support of the treatments, a psychological care adapted to the specificities of each patient should be implemented. This study should be completed and enriched by other research to bring real recommendations in terms of public health for these young patients in their care course. Concerning the impact and the financial burden of these diseases, it would be interesting to conduct medico-economic evaluation studies and to evaluate the costs generated in a macro and micro-economic dimension of these diseases. In addition, it would be interesting to imagine interventional studies to create levers to improve children’s psychosocial skills and to measure what could be done to improve the psychological care of patients and their caregivers.

## Additional file

Additional file 1: Full Patients’ Verbatim Comments. (DOCX 19 kb)

## Data Availability

The datasets during and/or analyzed during the current study are available from the corresponding author on reasonable request.

## Abbreviations

ALD: Long Term Disease
CMUc: Universal social coverage
OHRQoL: Oral health-related quality of life
RD: Rare disease
