# Supplementary material for: Oral health related quality of life of children and adolescents affected by rare orofacial diseases: a questionnaire-based cohort study
Source: Orphanet J Rare Dis. 2019 Jun 4;14:124. doi: 10.1186/s13023-019-1109-2 (PMC6549379; doi:10.1186/s13023-019-1109-2)
Supplement: Supplementary file 1 — Full Patients’ Verbatim Comments. (DOCX 19 kb) [file 13023_2019_1109_MOESM1_ESM.docx]

**Additional file:**

**FULL PATIENTS’ VERBATIM COMMENTS:**

Topic 1: Relations with caregivers and hospitals: patients reported a lack of information on the treatment and the course of care, a lack of listening from medical and non-medical staff, not enough psychological care of children and their parents. They also reported complicated relations between care in public hospitals and care in private establishments.

-“ *… Little consideration of the psychological side. Numerous difficulties and administrative delays in obtaining support...”*(multiple agenesis)

- “*It took 9 years for a dentist to agree to take her in consultation and only after an initial try. Until then we had to travel 220 km for M. to have dental care. We know that in the case of the care to be given (caries or other treatment), it will be necessary to return to the hospital so that he can benefit from sedation”*(autism)

-“*It would be interesting to propose in parallel with surgical and oral care, the accompaniment of interviews with psychologists so that the children can, if the need arises, evoke their difficulties of daily life and their possible feelings of being "different"*. (lip cleft)

-“*My daughter was born with a cleft palate. Care is complicated to give in relation to her intellectual disability. There is a lack of information about the slit and its support from a specialist (speech therapist) from the youngest age and a lack of competent specialists. His care has been given so far under MEOPA but with 3 people to hold her despite the anxiolytic drug given before...*” (lip/palate cleft)

- “*the relationship with a city dentist in a competent hospital was very complicated for us. We had to look for ourselves on the internet so as not to leave my son without doing anything ...*” (dentinogenesis imperfecta)

-“ *… Well-informed parents will always do more! Is it freedom of care? But we no longer have the freedom to pay for care...”* (Down Syndrome)

Topic 2: Patients and their parents reported feelings of anxiety, loneliness and difficulties in facing the gaze of others. They expressed that altered smiling has consequences for self-confidence.

-“*Our daughter suffers from significant visible agenesis (only 9 definitive sharp teeth). After many consultations we realized that Elisa would need a bone graft on the mandible and maxilla for future implants. Of course, Elisa is 15 years old and would like to live her youth like all girls of her age, when the gaze of others is important…”* (Ectodermal dysplasia)

-“*Our child does not seem affected by this malformation, however he is an anxious little boy. Is it because of the operations?”* (lip/palate cleft)

-“*the dental agenesis of my children is on half of the incisors, it causes problems of insurance and smiling.”* (multiple agenesis)

-“*We just saw the doctor for my cleft palate and he told me that I had good growth and that I am pretty. That really pleased me.”* (lip/palate cleft)

Topic 3: Transportation, distance from home-care center: No or little support for transport, feelings that the best practitioners are far from home, parents are forced to take a day off for the medical appointments of their children

- “…. *Until then, we had to travel 220 km for Mr. to have dental care. We know that if you take care of it (caries treatment or other), you will have to go back to the hospital so that he can benefit from sedation.”* (autism)

- “*….. we do not take into account all the trips every 2 weeks to keep track (70 km each time) and the time it takes. I am obliged to have all my Wednesday afternoons free for the follow-up, so I could have a better salary without these follow-ups ..”* (palate cleft)

Topic 4: Patients reported many difficulties in the care course.

- “*Lack of intermediate solutions waiting for future implants. Little consideration of the psychological side. Difficulties / administrative delays in obtaining an ALD .... and then difficulties of reimbursement of hospital bills.”* (multiple agenesis)

- "*Our daughter suffers from significant visible agenesis (only 9 definitive sharp teeth). After many consultations we realized that Elisa would need a bone graft on the mandible and maxilla for future implants. Up to now, the treatment has not started, and we have doubts about the people able to carry out this major operation ... we are in a phase of doubts and unfortunately, no one is worried about our health or problems related to the health of our daughter. We would like to find the right health professional with the necessary skills and for who our daughter would not be just a case study or squeeze cash out of the patients by charging up to 160 euros for 20 minutes of consultation …*” (Ectodemal dysplasia).

- “*Not much is known about this disease, hence also the difficulties of adapted proper care …”* (Anydrotic ectodermal dysplasia)

Topic 5: Patients and their parents reported issues about school and educational integration: difficulties to integrate normal schools, school delay, and isolation from others. They reported that pediatric metal caps were very visible in the mouth and had many consequences on smiling.

- “*A certain distance and serenity in our little boy, greater maturity too, but he appears isolated from the others in the class, and he has daily difficulties eating, brushing his teeth properly*...” (Lip/palate cleft)

- “*For our son the anxiety is more related to the hole in his mouth, the apprehension of pain and embarrassment about speech for poems and English lessons…*” (Complete bilateral Lip/palate cleft)

-“*…* *Her learning delay is explained by the fact that she does not follow the same school program as the children who are normally in school. …*” (MIH)

-“..*our daughter lives her ex-slot relatively quietly (operated at 3 months and 5 years). A scar that she finds unsightly sometimes comes through the gaze of some who question her or when she looks at herself (mirror, photos). But finally, this remains quite infrequent. Some definitive teeth in front pushed almost perpendicularly. For 5 months she has worn a removable dental device (withdrawal for meals) which annoys her: it’s a problem to speak, she has to remove it before eating, to brush her teeth at the school, no candy ... She’s anxiously waiting for all her teeth to be straight and that her lip can be retouched…*” (Lip/palate cleft)

-“*… the others at school ask me questions about the spots that are on my teeth so I answer that I have an illness. I wish we could not see my spots and not have to pay attention to the temperature of what I eat*”. (Amelogenesis imperfecta)

*-“ ... I am well integrated now because I understood how it works at school. We must impose ourselves from the beginning and show that we are strong. If someone makes fun of my teeth I answer him in the same tone and if he becomes aggressive I also answer him in the same tone. And it works. For my illness I know that it will not be cured and I know that there is a thousand times worse. So, I accept it...”* (Amelogenesis imperfecta)

-“ *... What really bothered me was to miss the school and see 5 dentists who did not know what to do. It is my great grandfather who found the address of the Rothschild hospital where they take good care of me but it is far from home: I have to take the train and miss the school each time and it bothers me ...*” (severe oligodontia)

Topic 6: Patients reported daily life-dental issues: pain, difficulty when eating, brushing teeth, altered tooth color, reduced chewing surface, lack of saliva, restricted mouth opening.

-“ … *Fortunately for my daughter, the most affected molars were quickly protected by crowns, allowing her to eat, drink, and brush her teeth almost normally. Spots of color that do not interfere in any way in her everyday life affect the incisors. The dentist who cares her is very attentive to my daughter. She explains all the acts she does and keeps us informed of every possible evolution of her illness.”* (MIH)

- "... *With regard to the mouth and teeth, the main problem of Peter is the small opening of his mouth, which causes difficulties to be understood (speak, articulate), but he can sing easily. It’s difficult to provide dental care, daily care and dentists care, difficulty eating certain foods, use of some spoons ...* " (Cleft with WOLF-HIRSCHHORN syndrome)

-“ *...Regarding the disease, my daughter suffers from other points where the teeth have an impact on her everyday life - the lack of saliva (which on the one hand impacts her oral health but also her appearance, she always has white traces on the edge of the lips ... dry mouth) – she also has real problems with pronunciation and can’t say the letter “R” - the 2 upper canines are missing finally, with slow growth for 2 years ... chewing and eating is sometimes complicated - sleeping with your mouth open, breathing more through the mouth than through the nose, a factor that accentuates dry mouth ...*” (Down syndrome with multiple agenesis)

-“*L. had a cleft palate (operated in 2010), anarchic and yellow teeth, and anarchic teeth. L. does not eat anything by mouth, she is nourished exclusively by gastrostomy, as well as hydrated by it. She is autistic, she does not speak at all and therefore does not show many signs of pain. It is I who am aware if her pains come from the mouth or not. Dental care is very complicated because L. does not understand what is said to her and cannot bear to be touched on her mouth...*” (Rapp Hodgkin Syndrome)

*-“ ... it makes me feel bad when I brush my teeth. The front teeth they move forward slowly and it bothers me. The others at school ask me questions about the spots on my teeth so I answer that I have an illness. I wish we could not see my spots and no longer have to pay attention to the temperature of what I eat...”* (Amelogenesis imperfecta)

Topic 7: Many patients reported budget and financial issues: to many types of healthcare are poorly or not at all reimbursed, poor reimbursements for orthodontic care. Patients reported their deception about social welfare coverage: problem of excess fees, feeling that it is "shameful to have no help"

-“… *it is an enormous budget to have my son's milk teeth cared for. The laying of the fluoride varnish is not supported by the social security for my son while it is necessary to maintain his milk teeth and final teeth to the maximum, even if eventually implants will be needed in adulthood. So as social security does not support this care, my private insurance reimburses me nothing either ...*” (Amelogenesis imperfecta)

–“.. *It is sometimes difficult to deal with all these practitioners and their rates because we still have unpaid orthodontics costs. The orthodontic care is not always as painful as it seems... My son sometimes has trouble with the secondary consequences of his malformation and in particular the trans tympanic aerators that he will have to keep for life because the tests of living without have failed…”* (Lip/palate cleft)

-“*Social security should make an effort to repay the implants needed by children.”* (lip/palate cleft)

-“*For the moment the disease of my daughter (my son is also 3 years, too) does not pose any particular concern, we are well followed-up; she takes it well because it’s not too embarrassing except to chew. Problems and support may come with age. I already spend a lot of money not taken care of ... I worry about the future with my 2 children affected by this disease*…” (dentinogenesis imperfecta)

- “*When it comes to orthodontic treatment, it's a big financial worry for us parents*.” (complete bilateral lip/palate cleft)

-“*No financial help or to help in the steps or help to take or know or make appointments with specialists. Even at the health insurance fund we are sent to walk in the desert. I find it shameful that we are not helped and that there is no help ...”* (ectodermal dysplasia)

- “*A care path is more like a search path. We lack benchmarks, guides and especially equality in treatment. Well-informed and well-informed parents will always do more! Is it freedom of care? But we no longer have the freedom to fund care...”* (Down syndrome)

Topic 8: Patients and most parents reported anxiety and fear about the future. They also expressed their doubts about the best practitioners to treat their children. Children reported anxiety related to worry about pain. Parents reported their impression of “fighting alone”, and their fear of the impact of teeth on overall health.
